# Research on the Identification of Road Hypnosis Based on the Fusion Calculation of Dynamic Human–Vehicle Data

**DOI:** 10.3390/s25092846

**Published:** 2025-04-30

**Authors:** Han Zhang, Longfei Chen, Bin Wang, Xiaoyuan Wang, Jingheng Wang, Chenyang Jiao, Kai Feng, Cheng Shen, Quanzheng Wang, Junyan Han, Yi Liu

**Affiliations:** 1 College of Electromechanical Engineering, Qingdao University of Science and Technology, Qingdao 266000, China; zhanghan@mails.qust.edu.cn (H.Z.); chenlongfei@mails.qust.edu.cn (L.C.); wangbin@mails.qust.edu.cn (B.W.); jiaochenyang@mails.qust.edu.cn (C.J.); fengkai@mails.qust.edu.cn (K.F.); b024030003@mails.qust.edu.cn (C.S.); 0020030005@mails.qust.edu.cn (Q.W.); hanjunyan@mails.qust.edu.cn (J.H.); yiliu@mails.qust.edu.cn (Y.L.); 2 Department of Mathematics, Ohio State University, Columbus, OH 43220, USA

**Keywords:** road hypnosis, driver, human–vehicle, heterogeneous data, fusion calculation, vehicle

## Abstract

Driver factors are the main cause of road traffic accidents. For the research of automotive active safety, an identification method for road hypnosis of a driver of a car with dynamic human–vehicle heterogeneous data fusion calculation is proposed. Road hypnosis is an unconscious driving state formed by the combination of external environmental factors and the psychological state of the car driver. When drivers fall into a state of road hypnosis, they cannot clearly perceive the surrounding environment and make various reactions in time to complete the driving task. The safety of humans and cars is greatly affected. Therefore, the study of the identification of drivers’ road hypnosis is of great significance. Vehicle and virtual driving experiments are designed and carried out to collect human and vehicle data. Eye movement data and EEG data of human data are collected with eye movement sensors and EEG sensors. Vehicle speed and acceleration data are collected by a mobile phone with AutoNavi navigation, which serves as an onboard sensor. In order to screen the characteristics of human and vehicles related to the road hypnosis state, the characteristic parameters of the road hypnosis in the preprocessed data are selected by the method of independent sample T-test, the hidden Markov model (HMM) is constructed, and the identification of the road hypnosis of the Ridge Regression model is combined. In order to evaluate the identification performance of the model, six evaluation indicators are used and compared with multiple regression models. The results show that the hidden Markov-Ridge Regression model is the most superior in the identification accuracy and effect of the road hypnosis state. A new technical scheme reference for the development of intelligent driving assistance systems is provided by the proposed comprehensive road hypnosis state identification model based on human–vehicle data can provide, which can effectively improve the life recognition ability of automobile intelligent cockpits, enhance the active safety performance of automobiles, and further improve traffic safety.

## 1. Introduction

In the 1930s, researchers conducted research on the phenomenon of “sleeping with eyes open” during driving and wrote a research report entitled “Sleeping with the Eyes Open” [1], which point out that car drivers may fall into a strange state of “sleeping with their eyes open” under certain circumstances. According to the survey, fatal accidents caused by single-car accidents account for about one-third of traffic fatalities in the United States, of which 50% are caused by fatigue, distraction, and other personal causes [2]. When driving on a calm road or highway, there are almost no features that can keep people alert, which easily puts the driver in a hypnosis state [3,4,5,6]. Williams G.W. et al. [7] described the phenomenon of a hypnosis-like state due to long-term driving in the monotonous environment of the highway. They pointed out that it is a state of amnesia, trance, and slow reaction speed, where the drivers can still drive normally; they also pointed out the difference between this phenomenon and sleep. Williams G.W. et al. [8] further induced this phenomenon on the monotonous highway by letting drivers focus on the highlights. They described it as a state of drowsiness, distorted thoughts, and judgments. Wertheim A. M. [9] proposed the eye movement theory: it is very easy to cause this hypnosis-like phenomenon when the driver has to watch these objects for a long time, and these objects move in a predictable mode within the driver’s visual range. Cerezuela G.P. et al. [10] believed that in this state, the driver is in an unconscious driving mode. When the drivers are in an abnormal state, such as hypnosis, their own physiological and psychological characteristics will change accordingly. Larue G.S. [11] found through their research that the drivers’ low alertness when they are in a hypnosis-like driving state can be evaluated by electroencephalogram (EEG). The researchers designed a virtual driving experiment to induce the driver to produce this hypnosis-like phenomenon by designing a monotonous road environment and used EEG to evaluate the driver’s low alertness when they were in a hypnosis-like driving state. It was found that the decline in the drivers’ alertness is caused by the monotony of road design and road scenery. In this state, the driver’s lane positioning, lane crossing time, blinking frequency, etc., can be affected to varying degrees.

The predecessors had some studies on hypnosis-like driving state. However, this hypnosis-like driving state is only explained as a phenomenon by these studies. Road hypnosis was not defined before, and research related to road hypnosis identification had not been carried out before. Based on this, Xiaoyuan Wang et al. [12] carried out in-depth research on road hypnosis. They defined road hypnosis as an unconscious driving state formed by the comprehensive effect of external environmental factors and the psychological state of the driver. Although the driver in this state seems to be able to maintain a normal driving state, the reaction speed is obviously slower than in the normal driving state. On the basis of clarifying the definition of the road hypnosis, Xiaoyuan Wang induced drivers to create a road hypnosis state and collected relevant data by designing and carrying out reasonable vehicle and virtual driving experiments. Eye movement characteristics, electrocardiogram characteristics, and electromyometric characteristics data are collected for the preliminary exploration of road hypnosis. The essential characteristics of road hypnosis were deeply explored through the use of electroencephalogram data [13]. The identification model of road hypnosis based on the driver’s physiological characteristics was initially established by integrating the characteristics of electroencephalogram data and eye movement data [14]. However, the shortcomings of the existing studies are as follows: the important role of vehicle parameters in the identification of the hypnosis state has not been taken into account, and there is no road hypnosis state identification model based on the synthesis of human and vehicle parameters.

Based on this, human and vehicle data of drivers in the state of road hypnosis are collected by designing and carrying out vehicle and virtual driving experiments. The independent parameter T test method is used to test the significance of human data and vehicle data, and extract characteristic parameters related to road hypnosis. Based on the hidden Markov algorithm and the Ridge Regression algorithm, a road hypnosis state identification model based on the fusion calculation of car dynamic human–vehicle multi-source heterogeneous data is built to achieve accurate identification of road hypnosis state in this study. The innovations of this study are as follows:

(1) A computational method based on multi-source heterogeneous data is used to fuse and calculate a variety of physiological parameters for identifying road hypnosis.
(2) Vehicle data are collected as the input of the model, and the influence of vehicle data on the recognition of the driver’s road hypnotic state is explored.
(3) Vehicle data and physiological data are fused and calculated, which provides a new method for real-time identification of road hypnosis.

## 2. Related Works

In existing research, driver physiological data and vehicle data can be used to identify abnormal driver behavior. The support vector machine (SVM) algorithm combined with the multimodal biometric recognition method was used by Zhou S. [15] et al. to identify driver fatigue and high-pressure driving behavior. The combination of decision trees and support vector machines (SVM) was used by Sun L. [16] to fuse physiological data (ECG and EDA) with vehicle data (vehicle speed, acceleration) to analyze the driver’s emotional state and identify the driver’s road rage driving behavior. Convolutional Neural Networks (CNNs) and long short-term memory networks (LSTMs) were used by Xiang G. [17] to analyze drivers’ facial expressions and physiological signals and detect drivers’ emotional changes in combination with vehicle data. LSTMs are used to analyze physiological signals in time series, and abnormal driving behaviors of drivers under emotions such as anger, anxiety, or fatigue can be identified in this study. The feature fusion method combining multi-layer perceptron (MLP) and the Bayesian Ridge Regressionsupport vector machine (SVM) was used by Park S. [18] to distinguish the driver’s fatigue driving, distracted driving, and high-stress driving status in real time. Physiological signals (electrocardiogram (ECG) and electroencephalogram (EEG)) and vehicle data (speed, acceleration, accelerator pedal pressure, brake strength, etc.) are fused in a weighted manner. Convolutional neural networks (CNNs) and deep belief networks (DBNs) were used by Tan D et al. [19] to classify abnormal driving behaviors and identify distracted driving behaviors. The fusion calculation of weighted support vector machine (WSVM) and neural network (NN) algorithm was used by Qu Y. [20] to monitor the driver’s status. WSVM is used to weigh the importance of different data sources. NN is used to judge the driver’s fatigue and distraction status. Neural networks were used by Wang L. [21] to process EEG, ECG, and vehicle data to identify whether the driver is in a fatigued stat. Although the above examples are available for identifying abnormal driving behaviors of drivers, there is no research that can identify road hypnosis through the fusion calculation of dynamic human–vehicle data. The theoretical basis is provided by these studies for this research.

## 3. Methodology

### 3.1. Road Hypnosis

Road hypnosis is defined as an unconscious driving state formed by the combined effect of external environmental factors and the driver’s own psychological state. Unlike fatigue driving, it is caused by repetitive and low-frequency stimulation in a highly predictable driving environment. Its specific manifestations are the driver’s perception paralysis, decreased attention, decreased vigilance, and is accompanied by temporary trance, amnesia, and fantasy. Once the drivers are out of the road hypnosis, they are usually accompanied by a clear alert state. Drivers often do not remember what happened when they were hypnotized on the road, but they have a clear memory of the fainting state they have just experienced. In this state, although the drivers seem to be able to maintain a normal driving state, the reaction speed is obviously slower than in the normal driving state.

### 3.2. Experiment

#### 3.2.1. Induction and Data Acquisition Methods of Road Hypnosis

In this research, the induction method of road hypnosis is as follows:

(1) In terms of the selection of drivers, drivers who are more prone to road hypnosis are selected for experiments in this study. Because novice drivers drive vehicles relatively seriously and are not prone to road hypnosis. Novice drivers may be nervous or serious while driving, which will affect the experimental results. In order to reduce or eliminate this effect, this study did not select novice drivers as research subjects. It is required that the driver has more than 8 years of driving experience. The driver is required to be between 26 and 60 years old, physically and mentally healthy, with relatively fixed sleep habits and sleeping time points. In order to avoid differences in results due to gender, all drivers recruited in this experiment are male. A total of 45 men are selected as drivers in the experiment
(2) In terms of the selection of experimental scenarios, scenes with relatively simple road geometry need to be selected for the vehicle driving experiment and the virtual driving experiment. Based on previous research results [16], drivers’ road hypnosis is more likely to be induced by monotonous environments, such as tunnels and highways. This is because the design of highways usually pursues smoothness and high speed, so the road layout often lacks complex visual stimulation and changes. Due to the closure and unique light changes, tunnels can produce multiple sensory stimulations to the driver in a short period of time. These factors make it easier for drivers to be in a state of road hypnosis when driving in such an environment. Therefore, two scenarios, highway and tunnel, are selected to carry out the vehicle driving experiment and virtual driving experiment in this study.
(3) In the process of vehicle driving and virtual driving experiments, the experimental assistant observes the subject’s eye movement, electroencephalogram, electrocardiogram, and other information collected by eye movement equipment, EEG equipment, and human-factor equipment in real time. The relevant data, when the driver’s eye movement gaze point is concentrated in the center of the road ahead, and the EEG, electrocardiogram, and electrocardiogram characteristics are stable, are recorded. The experimental assistant immediately asks whether the driver was just in a state of road hypnosis after the driver is out of this state, and records the response, so as to build a road hypnosis state driving data with such data. Because during the driving process, the road hypnosis state is presented as an intermittent and discontinuous multiple appearance, rather than a long-term stable and continuous state, the constructed road hypnotic state data set is stitched together from multiple time series data, not a long-term continuous data. At the same time, the conventional driving data in the non-road hypnosis state is selected as the normal driving data set.
(4) After the data set is screened, the relevant researchers in the laboratory manually evaluate the selected data set and screen the collected data according to video playback. To organize and classify the experimental data, colleagues with relevant research experience in the laboratory utilize the recorded time points and periods from the experiment, and manually screen the ECG signals recorded during the experiment. The experimental data with typical road hypnosis characteristics is selected to make the road hypnosis data set. Compared to normal driving conditions, the gaze points of drivers in road hypnosis are more concentrated, the fixation durations are longer, and the blink durations increase in terms of eye movement characteristics. Compared to normal driving conditions, the heart rate variability of drivers is reduced, and the driver has a lower and more regular heart rate in terms of bioelectric characteristics. Similarly, the data with abnormal driving states is used to make the normal driving data set. During the experiment, individual subjects may be in a fatigued driving state due to physical consumption, irregular driving posture, and other factors. The data related to such situations will be eliminated. Finally, the road hypnosis data set and the normal driving data set are filtered out. The detailed method for determining the road hypnotic state can be found in reference [12].

#### 3.2.2. Scenes and Equipment

**Figure 1:**
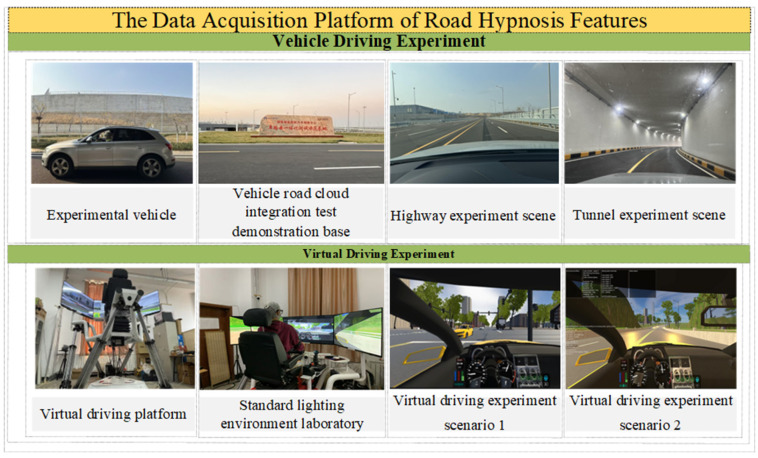
The data acquisition platform of road hypnosis features.

In this study, the vehicle and the virtual driving experiment are used to collect data, respectively. The vehicle driving experiment is carried out at the National Intelligent Network Innovation Center in Zhaoyuan City, Shandong Province, China-Vehicle-Road Cloud Integration Test Demonstration Base. The virtual driving experiment is carried out on the virtual driving experimental platform in the laboratory. The experimental vehicle used in the actual vehicle test, the actual vehicle driving experiment, and the virtual driving experiment environment are all shown in Figure 1. The data acquisition platform of road hypnosis features is shown in Figure 1. The tunnel is designed as a two-way, two-lane urban trunk road, with the left and right lines separated. The cross-section of the main tunnel is oval. There is a monotonous and closed straight-line driving distance. The highway is a two-way, four-lane road. The driving environment is relatively singular.

**Figure 2:**
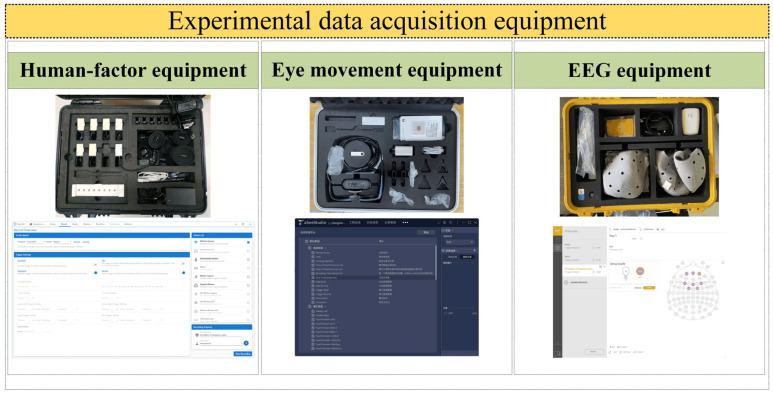
The experimental equipment.

The experimental equipment includes multi-functional comprehensive experimental test vehicles, virtual driving experimental platforms, EEG equipment, eye movement equipment, human-factor equipment, phones, laptops, and video recorders, etc. The experimental equipment is shown in Figure 2, which includes the display diagram of EEG equipment, eye movement equipment, and human-factor equipment. The EEG equipment used is Enobio Dx EEG data acquisition equipment. The manufacturer of this equipment is Neuroelectrics. This is a company based in Barcelona, Spain. It can collect eight channels of EEG data. The eye movement equipment used is a See Glasses eye tracker. Its specific parameters are shown in the table.

#### 3.2.3. The Procedure of Experiment

(1) Vehicle driving experiment

The vehicle driving experiment is carried out from 9:00 a.m. to 12:00 a.m. Before the experiment, the subjects are required to maintain adequate sleep. In addition to the experimental subjects, four experimental assistants participated in the vehicle driving experiment. In order to ensure the safety of the subjects, it is required to keep the driving speed at 60 km/h as much as possible and to maintain a uniform speed. Subjects are required to keep driving in a straight line as much as possible, under the condition of ensuring safety. The specific experimental process is as follows:

a. Before the experiment, an experimental assistant puts on eye movement equipment and the EEG equipment for the subjects and connects them to the laptop. At the beginning of the experiment, the experimental assistant is responsible for recording the start time and total duration of the experiment.
b. The experiment begins at the specified time, and the subjects drive the vehicle from the starting point of the tunnel or highway to the end point. During this journey, an experimental assistant is responsible for observing the road conditions and ensuring the safety of the subjects. The other two experimental assistants are responsible for collecting the road hypnosis status data of the subjects. After reaching the finish line, subjects are required to rest for 15 min. During this period, the experimental assistants disassemble the equipment, debug the experimental equipment, check the power of the equipment, and collect other information. An experimental assistant takes the initiative to ask the subject if they felt a road hypnosis in the driving process and records it.
c. Repeat the above experimental process. After all the experimenters collect the data, sort out the experimental equipment and end the experiment.

(2) Virtual driving experiment

The virtual driving experiment is carried out from 9:00 a.m. to 12:00 a.m. In addition to the subjects, three other experimental assistants participated in the virtual driving experiment. The specific experimental process is as follows:

a. Before the experiment, an experimental assistant is responsible for debugging the equipment and putting on the required equipment for the subjects. Subjects board the virtual driving platform. At the beginning of the experiment, the experimental assistant is responsible for recording the start time and overall duration of the experiment.
b. In the experiment, subjects perform driving tasks as required. They are required to keep the vehicle speed at 120 km/h. They are not allowed to change lanes in the middle. They must turn back when driving to the end. During the experiment, two experimental assistants are responsible for collecting the road hypnosis status data of the subjects until the end of the virtual driving mission section.
c. After a single experiment, an experimental assistant takes the initiative to ask the subject whether there are abnormal behaviors such as hypnosis and sleepiness during the driving process, records the subjects with abnormal behaviors, and asks whether the subjects are aware of entering road hypnosis, and records the answers. During this period, an experimental assistant is responsible for inspecting and debugging the equipment. The subjects exit the virtual driving platform. The experimental assistant helps the subjects take off the experimental equipment.
d. The above experimental process is repeated until all subjects complete the experiment. After the experiment is completed, the experimental equipment is sorted, and the experiment ends.

Through the induction and data acquisition methods of road hypnosis mentioned in Section 3.2.1, the data of 5 subjects in the vehicle driving experiment are excluded, and the data of 5 subjects in the virtual driving experiment are also excluded. Finally, 40 sets of vehicle driving experiment databases and 40 sets of virtual driving experiment databases are screened out.

### 3.3. Model and Algorithms

**Figure 3:**
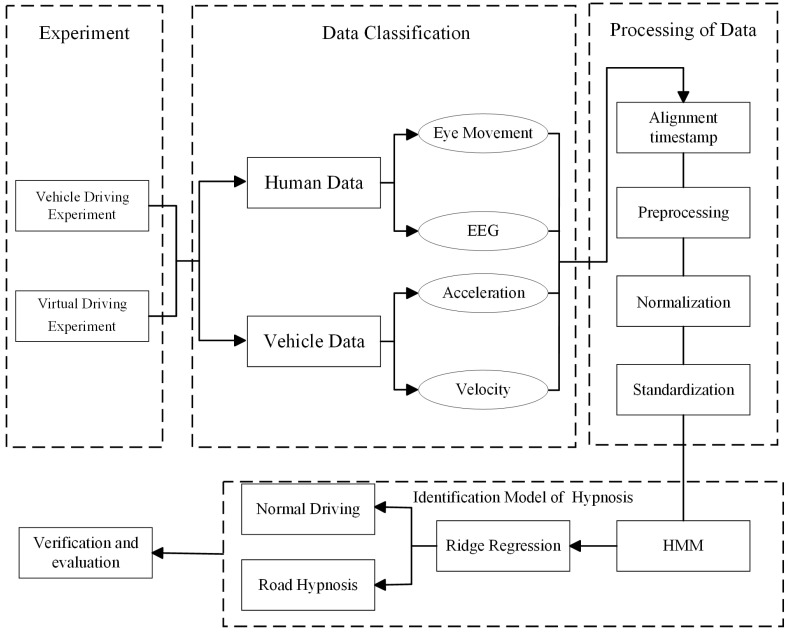
Road hypnosis state identification algorithm process.

Figure 3 shows the whole process of realizing road hypnosis identification. A road hypnosis state identification algorithm based on the fusion calculation of dynamic human–vehicle heterogeneous data are proposed in this study. After normalizing and standardizing the physiological data and vehicle data of the driver in the road hypnosis state, a road hypnosis state identification model based on the hidden Markov model is constructed.

The hidden Markov model (HMM) [22] is a probability-based statistical model that is widely used in sequence data modeling and time series prediction. Dependencies in time series data can be captured by it. The basic idea is to explain the observation data through a series of hidden states that cannot be directly observed. The driver’s road hypnosis state is an implicit state that cannot be directly observed. This state cannot be directly observed, but it can be indirectly inferred through physiological data and vehicle data. Physiological data and vehicle data can be jointly modeled as observation variables. However, a single HMM may have a fitting problem in high-dimensional data. The generalization ability of the model can be improved by introducing Ridge Regression. The complexity of the model can be effectively controlled by Ridge Regression. Over-fitting by adding L2 regularization terms to the loss function can be avoided [23]. The modeling advantages of the hidden Markov model for complex timing data can not only be utilized by the combination of the two, but the model performance can also be optimized through the regularization characteristics of Ridge Regression. Therefore, in this study, the HMM and Ridge Regression combination algorithm are selected to build an identification path hypnosis model based on human–machine multi-source heterogeneous data fusion.

#### 3.3.1. Data Preprocessing Method

(1) Original data types and data processing methods

Two types of data are collected in this study, namely the driver’s human data and vehicle data. The driver’s human data are divided into eye movement data and EEG data. The preliminary processing methods of the two types of data are as follows. Eye movement equipment parameters and their meanings are shown in Table 1.

a. Data collection settings: The EEG data channels selected in this study are all located in the frontal lobe area. The Fp2, Fpz, Fp1, F4, Fz, F3, FC2, and FC1 channels are selected. The basis for selecting these channels is based on the previous research of the reference team [13]. The EEG sampling rate is set to 500 Hz. The sampling rate of eye movement data and vehicle data is set to 100 Hz. In order not to affect the data of each frequency band of EEG data, the EEG data filtering range in this study is 0.2 to 60 Hz.
b. Data collation: After the data collection is completed, the EEG, eye movement data, and vehicle data obtained from vehicle driving and virtual driving experiments are sorted out. Forty groups of data are collected. EEG data are positioned by electrodes, bilateral mastoid average reference, filtering processing [24]. Eye movement data are initially screened and filtered [25], and vehicle data are filtered [26].
c. EEG

The EEG signal (EEG) is a time series signal with significant time change. In the analysis of EEG data, in order to capture the signal characteristics on different time scales, the sliding window method is applied to segment the analysis of the signal [27]. The sliding window is a method of defining a fixed-length window in the signal and continuously sliding the window along the timeline to gradually extract the local characteristics of the signal. In each window, the frequency band power in that period is calculated to capture the changes in the signal on different time scales. We set the original EEG signal as x(t), where t∈[0,T], T is the total duration of the signal. By sliding the window, the signal is divided into multiple windows with a length of W and gradually slides through the step length S. The spectrum analysis of each window is calculated by Power Spectral Density (PSD). For window i, the signal in the window is $x_{i}(t)$. The value of the power spectrum density $P_{x_{i}}(f)$ of the window at f frequency can be calculated by the Fourier transform (FFT) [28]:

(1) $$P_{x_{i}}(f)=\frac{1}{T_{f}}{\left|F\{x_{i}(t)\}\right|}^{2}$$

$F\{x_{i}(t)\}$ is the Fourier transform of the window signal, $T_{f}$ is the time resolution of the spectrum. Then, the power of each frequency band is calculated according to the predefined frequency band range. Power P can be expressed as the following:

(2) $$P=\int_{a}^{b}P_{x_{i}}(f)df$$

a and b are the upper and lower limits of *α*, *β*, *γ*, *θ,* and *δ* wave frequencies, respectively.

In order to ensure that the EEG data are aligned with the eye movement data and vehicle data on the time stamp, and to ensure that their time interval is consistent, the sliding window step length of the EEG data should be the same as the time interval of the eye movement data. Since the sampling rate of the eye movement data is 100 Hz and the time interval of the eye movement data is 0.01 s, the step length should be

(3) $$S_{sample}=0.01\times f_{EEG}$$

Therefore, $S_{sample}$ is set to 5 samples, where $f_{EEG}$ is the EEG sampling rate, and the window length is 1 s.

d. Eye movement data

Based on the previous study [12], four parameters of the eye movement data are selected in this study: Gaze Velocity, Pupil Diameter Left, Pupil Diameter Right, and IPD (Inter Pupillary Distance).

e. Vehicle data

In the virtual driving experiment, the vehicle data are the real-time output data of the virtual driving platform, including vehicle speed and acceleration. In the vehicle driving experiment, a car data acquisition device based on Gaode navigation, developed by our research group, is adopted in this study. Only a mobile phone is needed to collect the dynamic parameters of the car in real time during the driving process [29].

(2) Data normalization and standardization

The effective integration of driver’s human data and vehicle data is one of the key steps to improve the accuracy of model prediction. However, because these data from different sources usually have different scales, scopes, and distributions, they may be biased and incorporated directly into the model due to the differentiation of the data. In order to eliminate these differences and ensure that the model can fairly learn the contribution of each feature, it is necessary to normalize and standardize the data. Normalization is a kind of data preprocessing technology [30]. Feature data are compressed into a specified range [−1, 1] so that features of different scales have the same importance. In the context of multi-source data fusion, drivers and vehicles usually have different quantitative frameworks. For example, the EEG data in the driver’s data may be in microvolts (μV), while the vehicle data (such as vehicle speed) may be in km/h. This difference in the framework may lead to the learning process of certain feature-dominated models. In the collected data, the original data of eye movement and vehicle data can be directly used for normalization. There is no need to process it like EEG data. The Min-Max scaling method is used in the process of normalization in this study. The specific formula is as follows:

(4) $$X_{\mathrm{norm}}=\frac{X-X_{\min}}{X_{\max}-X_{\min}}$$

*X* is the original data, $X_{\min}$ and $X_{\max}$ are the minimum and maximum values of this feature, respectively, $X_{\mathrm{norm}}$ is the normalized data. The result is in the range of [−1, 1]. Through this method, all features are compressed to the same scale to eliminate the scale differences between different features.

Standardization is another common data preprocessing method [31]. The data are converted into a standard normal distribution with an average of 0 and a standard deviation of 1. The formula of standardization is as follows:

(5) $$X_{\mathrm{std}}=\frac{X-\mu }{\sigma }$$

*X* is the original data, μ and σ are the mean and standard deviation of the feature, respectively, and $X_{\mathrm{std}}$ is the standardized data.

#### 3.3.2. Road Hypnosis State Recognition Algorithm and Model

**Figure 4:**
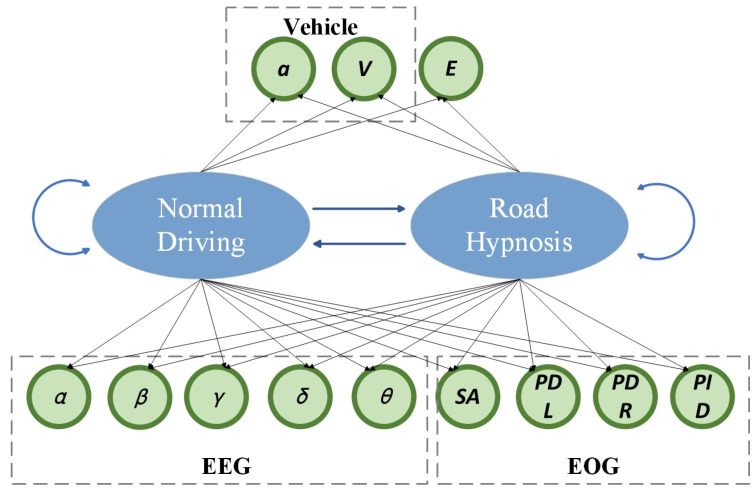
Schematic diagram of the hidden Markov model.

The combination of the hidden Markov model (HMM) and Ridge Regression is used to analyze multi-dimensional human and vehicle data to identify the driver’s road hypnosis. The hidden Markov model (HMM) is a probability model based on the Markov process used to describe an observation sequence generated by an implicit state sequence. Suppose there are two hidden states, which represent that the driver is in a road hypnosis state and a non-road hypnosis state, respectively. The goal of HMM is to infer the hidden state of each moment by observing data. The schematic diagram of the hidden Markov model is shown in Figure 4. The likelihood of road hypnosis versus normal driving is predicted by integrating vehicle data, eye movement data, and EEG data using a hidden Markov model (HMM), as illustrated by this diagram. In the vehicle box, a is acceleration, and velocity. In the EEG box, alpha, beta, gamma, delta, and theta brain waves are shown. In the EOG box, SA is Gaze Velocity, PDL is Pupil Diameter Left, PDR is Pupil Diameter Right, and IPD is Inter Pupillary Distance.

Formula of HMM is as follows:

(6) $$P(X,Y)=P(Y_{1})\prod_{t=2}^{T}P(Y_{t}\left|Y_{t-1}\right.)P(X_{t}\left|Y_{t}\right.)$$

$P(Y_{1})$ is the probability distribution of the initial state. $P(Y_{t}\left|Y_{t-1}\right.)$ is the state transfer probability, which represents the transfer probability from moment *t* − 1 to moment *t* state. $P(X_{t}\left|Y_{t}\right.)$ is the emission probability, which represents the probability of observing data $X_{t}$ when given a hidden state $Y_{t}$; X is the observation data sequence, Y is the hidden state sequence.

In this study, the hidden Markov model is trained by learning the state transfer matrix and emission matrix of the data. Through the training of the model, the state of each moment can be predicted, and the probability of each state can be calculated.

By calculating the probability of belonging to the road hypnosis state (state 1) at each moment, the indicator of “hypnosis degree” can be obtained, which is defined as follows:

(7) $${\mathrm{Hypnotic}\;\mathrm{Degree}}_{t\;}=P(Y_{t\;}=1\left|X_{t}\;\right.)$$

$Y_{t\;}=1$ indicates that the driver is in a hypnosis state, $P(Y_{t\;}=1\left|X_{t}\;\right.)$ is the probability that the driver is in a road hypnosis state after observing the data $X_{t}$ at the moment.

After obtaining the probability of the degree of road hypnosis, Ridge Regression is used to predict the degree of road hypnosis. Ridge Regression is a Linear Regression model that prevents over-fitting and solves the problem of multiple collinearities by adding L2 regularization terms. The target functions of Ridge Regression are as follows:

(8) $$\overset{\wedge }{\beta }=\arg\underset{\beta }{\min}\{\sum_{i=1}^{n}\left(y_{i}-X_{i}\beta \right)+\lambda \sum_{j=1}^{p}\beta_{j}^{2}\}$$

$\overset{\wedge }{\beta }$ is the estimated coefficient of the Ridge Regression model, β is the regression coefficient of the model, n is the observed sample, $y_{i}$ is the target variable (degree of hypnosis), $X_{i}$ is the characteristic vector, $\beta_{j}$ is the j-th regression coefficient, *λ* is the regression coefficient. Through Ridge Regression, multiple input features can be effectively used to predict the degree of road hypnosis. The complexity of the model can be controlled during the training process to avoid over-fitting.

The sign represented by the weight of each regression coefficient is shown in Table 2. The calculated formula for judging the hypnosis state of the road obtained is as follows:

(9) $$\mathrm{Hypnosis}\;\mathrm{Degree}=\sum_{i=1}^{14}x_{i}\beta_{i}+\beta_{0}$$

## 4. Extraction of Road Hypnosis State Characteristics

Before entering the driver’s human data and vehicle data into the hidden Markov model for road hypnosis identification, the purpose of conducting an independent sample *t*-test [32] is to screen out the data that makes a significant contribution to the model prediction. It can be determined whether there are significant differences between different sample groups by t-testing, so as to identify the characteristic variables that play a key role in the identification of road hypnosis states. Road hypnosis can be more effectively learned and identified by the model with the help of data of high significance, while the stability and accuracy of the model may be affected by noise introduced by data of low significance.

### 4.1. Human Characteristics of Drivers

In order to test the relationship between different drivers’ human data and road hypnosis states, whether road hypnosis is used as a group variable, and each parameter of EEG and eye movement data are used as a test variable, and an independent sample *t* test is carried out on the driver’s EEG data and eye movement data. The results are shown in Table 3 and Table 4, respectively.

**Figure 5:**
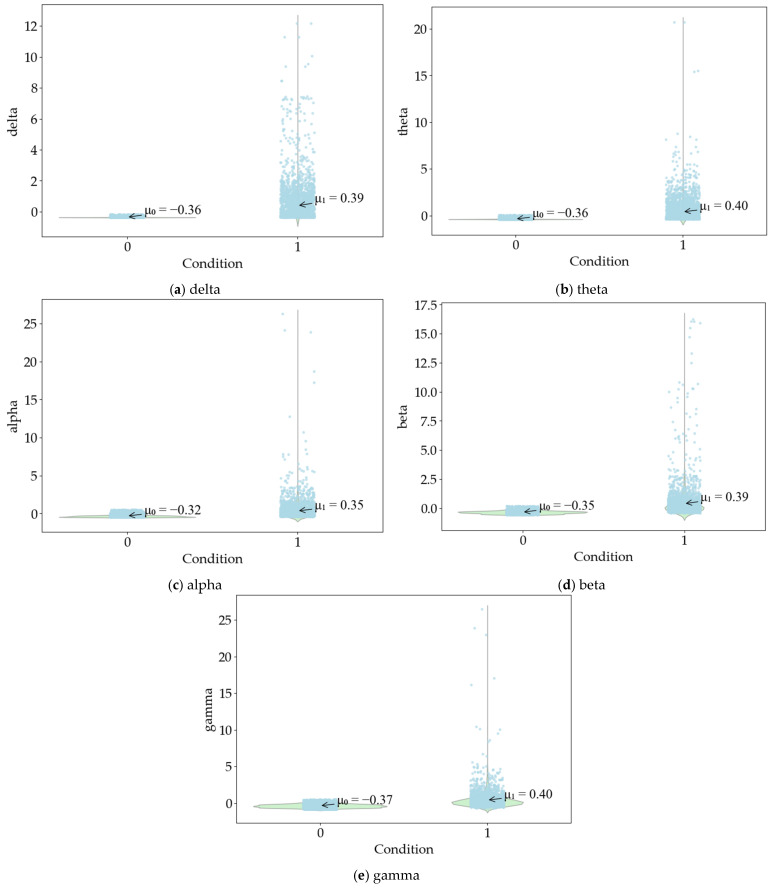
The distribution of EEG data.

The independent sample *t* test results of EEG data show that the delta value is less significant than 0.05 in the Levin variance equivalence test. Therefore, the heteroscedasticity assumption is selected. The mean equivalence *t* test sig. (2-tailed) value is 0.000, t = 28.711. There is a significant difference in whether the delta is in a state of road hypnosis. The theta value is less significant than 0.05 in the Levin variance equivalence test. Therefore, the heteroscedasticity assumption is selected. The mean equivalence *t* test sig. (2-tailed) value is 0.000, t = 29.051. There is a significant difference in whether the theta is in a state of road hypnosis. The alpha value is less significant than 0.05 in the Levin variance equivalence test. Therefore, the heteroscedasticity assumption is selected. The mean equivalence *t* test sig. (2-tailed) value is 0.000, t = 24.885. There is a significant difference in whether the alpha is in a state of road hypnosis. The beta value is less significant than 0.05 in the Levin variance equivalence test. Therefore, the heteroscedasticity assumption is selected. The mean equivalence *t* test sig. (2-tailed) value is 0.000, t = 28.124. There is a significant difference in whether the beta is in a state of road hypnosis. The gamma value is less significant than 0.05 in the Levin variance equivalence test. Therefore, the heteroscedasticity assumption is selected. The mean equivalence *t* test sig. (2-tailed) value is 0.000, t = 29.621. There is a significant difference in whether Gamma is in a state of road hypnosis. The distribution of EEG data is shown in Figure 5. The mean values of delta, theta, alpha, beta, and gamma in the road hypnosis state are 0.39, 0.40, 0.35, 0.39, and 0.40, respectively. The mean values of delta, theta, alpha, beta, and gamma in the normal driving state are −0.36, −0.36, −0.32, −0.35, and −0.37, respectively.

**Figure 6:**
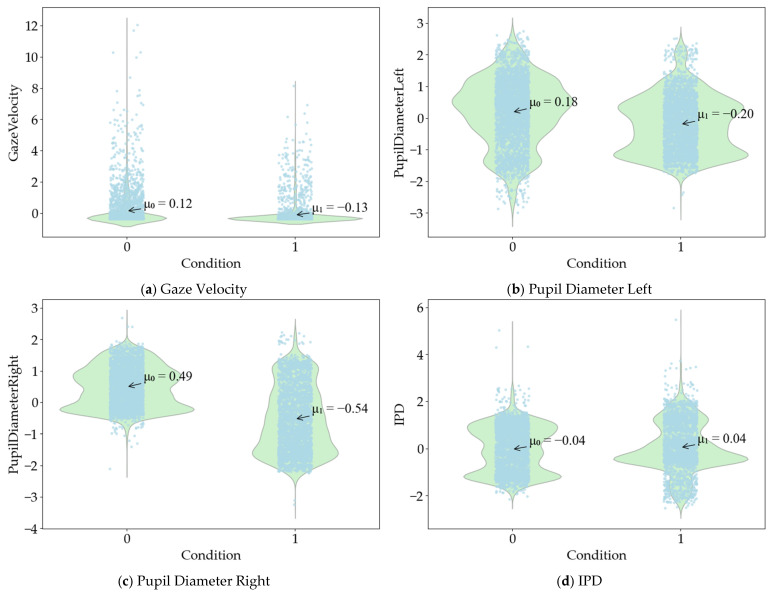
The distribution of eye movement data.

The independent sample *t* test results of eye movement data show that the Gaze Velocity value is less significant than 0.05 in the Levin variance equivalence test. Therefore, the heteroscedasticity assumption is selected. The mean equivalence *t* test sig. (2-tailed) value is 0.000, t = −9.722. There is a significant difference in whether the Gaze Velocity is in a state of road hypnosis. The Pupil Diameter Right value is less significant than 0.05 in the Levin variance equivalence test. Therefore, the heteroscedasticity assumption is selected. The mean equivalence *t* test sig. (2-tailed) value is 0.000, t = −43.440. There is a significant difference in whether the Pupil Diameter Right is in a state of road hypnosis. The Pupil Diameter Left value is less significant than 0.05 in the Levin variance equivalence test. Therefore, the heteroscedasticity assumption is selected. The mean equivalence *t* test sig. (2-tailed) value is 0.000, t = −14.631. There is a significant difference in whether the Pupil Diameter Left is in a state of road hypnosis. The IPD value is more significant than 0.05 in the Levin variance equivalence test. Therefore, the homoscedasticity assumption is selected. The mean equivalence T test sig. (2-tailed) value is 0.004, t = 2.898. There is a significant difference in whether the IPD is in a state of road hypnosis. The distribution of eye movement data are shown in Figure 6. The mean values of Gaze Velocity, Pupil Diameter Left, Pupil Diameter Right, and IPD in the road hypnosis state are −0.13, −0.20, −0.54, and 0.04, respectively. The mean values of Gaze Velocity, Pupil Diameter Left, Pupil Diameter Right, and IPD in the normal state are 0.12, 0.18, 0.49, and −0.04, respectively.

### 4.2. Vehicle Characteristics

In order to test the relationship between different vehicle data and the road hypnosis state, whether the road hypnosis is used as a group variable, each parameter of the vehicle data is used as a test variable. The vehicle data are tested independently. The result is shown in Table 5.

**Figure 7:**
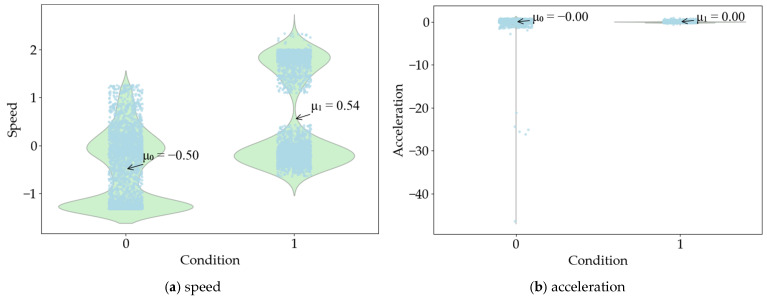
The distribution of vehicle data.

The independent sample *t* test results of vehicle data show that the speed value is less significant than 0.05 in the Levin variance equivalence test. Therefore, the heteroscedasticity assumption is selected. The mean equivalence *t* test sig. (2-tailed) value is 0.000, t = 44.698. There is a significant difference in whether the speed is in a state of road hypnosis. The acceleration value is less significant than 0.05 in the Levin variance equivalence test. Therefore, the heteroscedasticity assumption is selected. The mean equivalence *t* test sig. (2-tailed) value is 0.749, t = 0.320. There is no significant difference in whether the acceleration is in a state of road hypnosis. The distribution of vehicle data is shown in Figure 7. The mean values of speed and acceleration in the road hypnosis state are 0.54 and 0.00, respectively, and the mean values of speed and acceleration in the normal state are −0.5 and −0.00, respectively.

## 5. Results and Analysis

According to the independent samples t-tests conducted on drivers’ data and vehicle data, among the drivers’ data, the delta, theta, alpha, beta, and gamma waves of the EEG data, as well as the Gaze Velocity, left and right pupil diameters, and interpupillary distance (IPD) of the eye movement data, all show significant effects on whether the driver is in a state of road hypnosis. The data showing significant effects on road hypnosis states are selected as input data. A hidden Markov model (HMM)-based road hypnosis identification model is constructed. The road hypnosis state identification model is built using these inputs. The regression coefficient and intercept value of the output of the constructed model are shown in Table 6.

The value of intercept *β*_0_ is 0.349, and the values of *β*_2_, *β*_3_, *β*_11,_ and *β*_13_ are all 0.

Therefore, the calculated formula for the obtained road hypnosis judgment is as follows:

(10) $$\mathrm{Hypnosis}\;\mathrm{Degree}=\sum_{i=1}^{15}x_{i}\beta_{i}+0.349$$

During the training process, the data set is divided into a training set (70%) and test set (30%). Ridge Regression is used to predict the degree of hypnosis. The performance of the model is measured by the following evaluation indicators [33]: Mean-square error (MSE), coefficient of determination (R^2^), root mean square error (RMSE), Mean Absolute Error (MAE), explained variance (EV), and maximum error.

In this study, the standardized data are input into the regression model Linear Regression [34], Ridge Regression, Lasso Regression [35], Support Vector Regression [36] (SVR), Decision Tree [37], Neural Network [38], Bayesian Ridge Regression [39] model, and compare it with the road hypnosis identification model constructed in this study. The evaluation index results are shown in Table 7.

The result comparison plot is a commonly used regression model diagnostic diagram, which is used to evaluate the fitting effect of the model. The result comparison plot is used to evaluate how close the predicted values are to the true values. Ideally, the closer all points are to the red diagonal line, the more accurate the model’s predictions are. That is:

(11) $$Prediction\;Error_{i}={\overset{\wedge }{y}}_{i}-y_{i}$$

**Figure 8:**
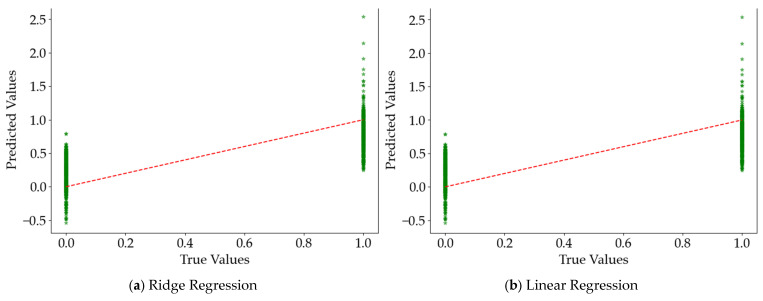

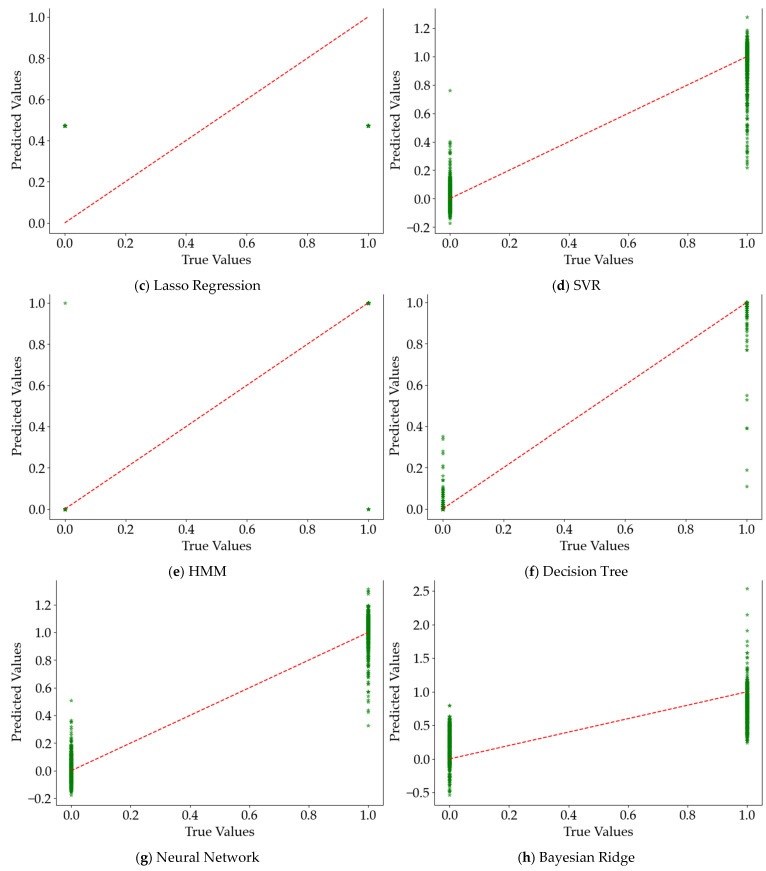
The result comparison plots for each regression model.

The result comparison plots for each regression model are shown in Figure 8. The green stars represent each observation, which is a scattered data point between two groups (0 and 1). The red dash line is the baseline that the model correctly predicted.

From the results of the independent samples *t*-tests conducted on driver characteristics and vehicle characteristics data in Section 3.1 and Section 3.2, it is shown that in eye movement characteristics, the Gaze Velocity, left and right pupil diameters, and interpupillary distance of drivers in a road hypnosis state exhibit significant change compared to those in a normal driving state. Among the EEG features, the driver’s δ, θ, α, β, and γ are significant in distinguishing whether the driver is in a road hypnosis state; among the vehicle features, the vehicle’s speed and acceleration are significant in distinguishing whether the driver is in a road hypnosis state. It can be seen from the regression coefficient results of the road hypnosis identification model based on the hidden Markov-Ridge Regression that in the eye movement characteristics, in the driver’s road hypnosis state, Pupil Diameter Left, Pupil Diameter Right characteristics are the most obvious, IPD (Inter Pupillary Distance) characteristic is more obvious, Gaze Velocity characteristic is relatively lower. In the EEG characteristic, the driver’s *δ*, *θ*, *α*, *β,* and *γ* in the road hypnosis state have significant changes compared with the normal driving state. It can be seen from the regression coefficient results of the road hypnosis identification model based on the hidden Markov-Ridge Regression that in the EEG characteristic, the driver’s road hypnosis state has the most obvious changes in the characteristics of *δ*, *θ* and *γ* characteristics, the *β* characteristic is more obvious, and the characteristic of *α* is relatively low. In the characteristics of the vehicle data, the regression coefficients of speed and acceleration are 0.111 and 0.105, respectively. It can be seen that vehicle data, speed, and acceleration contribute more to the road hypnosis recognition model and have a significant impact on road hypnosis recognition.

From the results in Table 7, the evaluation metrics of the regression models—Linear Regression, Ridge Regression, Lasso Regression, Support Vector Regression (SVR), Decision Tree, Neural Network, and Bayesian Ridge—are compared with those of the Hidden Markov-Ridge Regression model. It is shown that the constructed road hypnosis identification regression models all demonstrate good performance, with the Hidden Markov-Ridge Regression model performing the best. It achieves the lowest mean squared error (MSE) and Mean Absolute Error (MAE) among all models, as well as the highest coefficient of determination (R^2^) and explained variance (EV). Among the other regression models, the Decision Tree regression model also performs well, though slightly inferior to the Hidden Markov-Ridge Regression model. As shown in Figure 8, it can be seen that the HMM prediction has the most accurate values, and almost all 0 and 1 states (normal driving and road hypnosis) are correctly identified. It had fewer errors than the other models. It is demonstrated that the Hidden Markov-Ridge Regression model achieves the best fit between the true values and the predicted values.

## 6. Conclusions

This research is addressing the issue of road hypnosis identification for car drivers. By deeply analyzing driver characteristics and vehicle characteristics, an identification method for road hypnosis of car drivers with dynamic human–vehicle heterogeneous data fusion calculation is established. The core tasks of this research include the following:

(1) Vehicle and virtual driving experiments are designed and conducted. Data from 45 participants in both road hypnosis and normal driving states were collected. A road hypnosis state characteristic data set is established, which lays the foundation for the preprocessing and analysis of road hypnosis characteristic parameters and the construction of the road hypnosis identification model.
(2) Preprocessing methods for human data and vehicle data are introduced, which provide a cleaner and more standardized data basis for feature parameter extraction and model construction. The independent parameter *t*-test method is used to test the significance of driver characteristic data and vehicle characteristic data. Road hypnosis state characteristic parameters are extracted and used as input parameters for the subsequent road hypnosis identification model construction.
(3) A road hypnosis state identification model for car drivers based on Hidden Markov-Ridge Regression is constructed. To verify the effectiveness and accuracy of the model, it is compared and analyzed with regression models such as Linear Regression, Ridge Regression, Lasso Regression, Support Vector Regression (SVR), Decision Tree, Neural Network, and Bayesian Ridge Regression. Six evaluation metrics, including Mean Squared Error (MSE), Coefficient of Determination (R^2^), Root Mean Squared Error (RMSE), Mean Absolute Error (MAE), Explained Variance (EV), and Maximum Error, are introduced for model evaluation. The superiority of the constructed Hidden Markov-Ridge Regression model is verified.

A comprehensive human–vehicle integrated road hypnosis state identification model is proposed in this research, and a new technical solution for the development of intelligent driving assistance systems is provided. The life recognition capability of intelligent car cabins is effectively improved. The active safety performance of the vehicle is enhanced, and traffic safety is being further promoted.

A comprehensive road hypnosis state identification model based on humans and vehicles is not only proposed in this study, but a new technical scheme reference for the development of intelligent driving assistance systems is also provided. The life recognition ability of automobile intelligent cockpits is effectively improved, the active safety performance of automobiles is enhanced, and traffic safety is further improved. The limitation of this study is that the road hypnosis recognition model is only used for road hypnosis recognition in special scenarios, such as highways and tunnels, but drivers will also experience road hypnosis in daily driving environments. Research on this road hypnosis state identification is not involved in this research. In the future, the focus will be on the induction experiment design, data collection, and state identification research of road hypnosis, in general, environments such as daily driving.

## Figures and Tables

**Table 1:** Eye movement equipment parameters and their meanings.

Type	Name	Explanation
Pupil information	Validity Left	Validity of left eye pupil recognition: 0 means the recognition is successful; 1 means the recognition fails
	Validity Right	Validity of right eye pupil recognition: 0 means the recognition is successful; 1 means the recognition fails
	Pupil Diameter Left (mm)	Left eye pupil diameter
	Pupil Diameter Right (mm)	Right eye pupil diameter
	IPD (mm)	Interpupillary distance
Gaze point information	Gaze Point Index	Original gaze point index
	Gaze Velocity(px/s)	The speed at which the eyes move
	Gaze Point X (px)	Original fixation x-coordinate in pixels
	Gaze Point Y (px)	Original fixation y-coordinate in pixels
	Gaze Point Right X (px)	X-coordinate of the original gaze point of the right eye
	Gaze Point Right Y (px)	Y-coordinate of the original gaze point of the right eye
	Gaze Point Left X (px)	X-coordinate of the original gaze point of the left eye
	Gaze Point Left Y (px)	Y-coordinate of the original gaze point of the left eye
	Gaze Vector Left X	The x-coordinate of the left eye gaze vector
	Gaze Vector Left Y	The y-coordinate of the left eye gaze vector
	Gaze Vector Left Z	The z-coordinate of the left eye gaze vector
	Gaze Vector Right X	The x-coordinate of the right eye gaze vector
	Gaze Vector Right Y	The y-coordinate of the right eye gaze vector
	Gaze Vector Right Z	The z-coordinate of the left eye gaze vector

**Table 2:** Weight of regression coefficient.

Observation Sequence	Regression Coefficient	Observation Sequence	Regression Coefficient
Speed	*β*_1_	gamma	*β*_8_
Acceleration	*β*_2_	Gaze Velocity	*β*_9_
Delta	*β*_4_	Pupil Diameter Left	*β*_10_
Theta	*β*_5_	Pupil Diameter Right	*β*_12_
Alpha	*β*_6_	IPD	*β*_14_
Beta	*β*_7_	Intercept	*β*_0_

**Table 3:** *t*-test results of an independent sample of EEG data.

		Levin’s Variance Equivariance Test		Mean Equivalence *t* Test				
		F	Significance	t	Degree of Freedom	Sig. (2-Tailed)	Average Deviation	Standard Error
delta	Homoscedasticity assumption	1908.694	0.000	30.090	5538	0.000	0.751	0.025
	Heteroscedasticity assumption			28.711	2642.050	0.000 **	0.751	0.026
theta	Homoscedasticity assumption	1806.138	0.000	30.438	5538	0.000	0.758	0.025
	Heteroscedasticity assumption			29.051	2656.262	0.000 **	0.758	0.026
alpha	Homoscedasticity assumption	446.060	0.000	26.033	5538	0.000	0.661	0.025
	Heteroscedasticity assumption			24.885	2736.688	0.000 **	0.661	0.027
beta	Homoscedasticity assumption	486.039	0.000	29.443	5538	0.000	0.737	0.025
	Heteroscedasticity assumption			28.124	2698.158	0.000 **	0.737	0.026
gamma	Homoscedasticity assumption	307.435	0.000	30.904	5538	0.000	0.768	0.025
	Heteroscedasticity assumption			29.621	2884.348	0.000 **	0.768	0.026

Bold and ** indicate that the feature is significant.

**Table 4:** *t*-test results of the independent sample of eye movement data.

		Levin’s Variance Equivariance Test		Mean Equivalence *t* Test				
		F	Significance	t	Degree of Freedom	Sig. (2-Tailed)	Average Deviation	Standard Error
Gaze Velocity	Homoscedasticity assumption	192.138	0.000	−9.548	5538	0.000	−0.255	0.027
	Heteroscedasticity assumption			−9.722	5090.659	0.000 **	−0.255	0.026
Pupil Diameter Right	Homoscedasticity assumption	796.958	0.000	−44.350	5538	0.000	−1.025	0.023
	Heteroscedasticity assumption			−43.440	4336.223	0.000 **	−1.025	0.024
Pupil Diameter Left	Homoscedasticity assumption	54.213	0.000	−14.514	5538	0.000	−0.383	0.026
	Heteroscedasticity assumption			−14.631	5504.434	0.000 **	−0.383	0.026
IPD	Homoscedasticity assumption	1.791	0.181	2.898	5538	0.004 **	0.078	0.027
	Heteroscedasticity assumption			2.889	5401.695	0.004	0.078	0.027

Bold and ** indicate that the feature is significant.

**Table 5:** *t*-test results of an independent sample of vehicle data.

		Levin’s Variance Equivariance Test		Mean Equivalence *t* Test				
		F	Significance	t	Degree of Freedom	Sig. (2-Tailed)	Average Deviation	Standard Error
Speed	Homoscedasticity assumption	820.174	0.000	45.241	5538	0.000	1.040	0.0230
	Heteroscedasticity assumption			44.698	4940.143	0.000 **	1.040	0.0233
Acceleration	Homoscedasticity assumption	29.406	0.000	0.306	5538	0.760	8.230 × 10^−3^	2.690 × 10^−2^
	Heteroscedasticity assumption			0.320	2989.189	0.749	8.230 × 10^−3^	2.570 × 10^−2^

Bold and ** indicate that the feature is significant.

**Table 6:** The regression coefficient and intercept value of the output of the constructed model.

Observation Sequence	Regression Coefficient	Value	Observation Sequence	Regression Coefficient	Value
Speed	*β*_1_	0.111	Gaze Velocity	*β*_9_	−0.017
Delta	*β*_4_	0.097	Pupil Diameter Left	*β*_10_	0.093
Theta	*β*_5_	0.094	Pupil Diameter Right	*β*_12_	−0.235
Alpha	*β*_6_	0.033	IPD	*β*_14_	0.060
Beta	*β*_7_	−0.066	Acceleration	*β*_15_	0.105
Gamma	*β*_8_	0.089	Intercept	*β*_0_	0.349

**Table 7:** The evaluation index results.

Model	MSE	RMSE	MAE	R2	EV	Max Error
Linear Regression	0.100152	0.316469	0.257835	0.599232	0.59937	1.538337
Ridge Regression	0.100165	0.316488	0.257841	0.599184	0.599321	1.53835
Lasso Regression	0.250188	0.500188	0.499467	−0.00115	0	0.52685
SVR	0.019759	0.140565	0.088761	0.920935	0.922042	0.78287
Decision Tree	0.004513	0.067176	0.004513	0.981942	0.981946	1
HMM- Ridge Regression	0.003068	0.055387	0.010866	0.987724	0.987748	0.89
Neural Network	0.009819	0.099092	0.065211	0.960707	0.960733	0.701525
Bayesian Ridge	0.100244	0.316614	0.2579	0.598865	0.599006	1.537624

## Data Availability

The data presented in this study are available on request from the corresponding author. The data are not publicly available due to privacy.
